# Comparative Analysis of Circulating Levels of SARS-CoV-2 Antibodies and Inflammatory Mediators in Healthcare Workers and COVID-19 Patients

**DOI:** 10.3390/v14030455

**Published:** 2022-02-23

**Authors:** Luzia Maria de-Oliveira-Pinto, Victor Edgar Fiestas Solórzano, Maria de Lourdes Martins, Caroline Fernandes-Santos, Paula Hesselberg Damasco, Marilda Agudo Mendonça Teixeira de Siqueira, Helver Gonçalves Dias, Alex Pauvolid-Corrêa, Paulo Vieira Damasco, Elzinandes Leal de Azeredo

**Affiliations:** 1 Viral Immunology Laboratory, Instituto Oswaldo Cruz (IOC/Fiocruz), Rio de Janeiro 21040-360, Brazil; lpinto@ioc.fiocruz.br (L.M.d.-O.-P.); vicfiso@gmail.com (V.E.F.S.); carol.uned@gmail.com (C.F.-S.); helvergd@gmail.com (H.G.D.); 2 Rede Casa Hospital Rio Laranjeiras e Rio Botafogo, Rio de Janeiro 22240-000, Brazil; ccih@hospitalriobotafogo.com.br (M.d.L.M.); paulovieiradamasco@gmail.com (P.V.D.); 3 Departamento de Clínica Médica, Universidade Federal Fluminense (UFF), Niterói, Rio de Janeiro 242010-240, Brazil; paulahesselberg@hotmail.com; 4 Laboratório de Vírus Respiratório e Sarampo, Instituto Oswaldo Cruz (IOC/Fiocruz), Rio de Janeiro 21040-360, Brazil; mmsiq@ioc.fiocruz.br (M.A.M.T.d.S.); pauvolid@gmail.com (A.P.-C.); 5 Department of Veterinary Integrative Biosciences, Texas A&M University, College Station, TX 77843-4458, USA; 6 Faculdade de Ciências Médicas, Universidade do Estado do Rio de Janeiro, Rio de Janeiro 20550-170, Brazil; 7 Faculdade de Medicina, Universidade Federal do Estado do Rio de Janeiro, Rio de Janeiro 21941-902, Brazil

**Keywords:** SARS-CoV-2, COVID-19, healthcare workers, patients, antibodies, cytokines/chemokines, Brazil

## Abstract

Advances in knowledge of the pathophysiology of COVID-19 have been acquired; however, the host factors that could explain the mild and severe forms of the disease are not fully understood. Thus, we proposed to evaluate anti-SARS-CoV-2 antibodies and the inflammatory response of different groups of individuals, including healthcare workers (HCW), sick and dead COVID-19 patients and also recovered patients to contribute to this knowledge gap. Our objective is to relate the clinical evolution of these individuals with the level of detection and functionality of specific antibodies and with the production of inflammatory mediators. As main findings, IgA and IgG anti-SARS-CoV-2 were detected in asymptomatic HCW. IFN-γ and TNF-α levels were higher in symptomatic HCWs than patients with COVID-19 and those who died. Patients who died had higher levels of IL-6, IL-10, and CCL2/MCP-1. We found an imbalance between antiviral and pro-inflammatory mediators in the groups, in which IFN-γ and TNF-α seem to be more associated with protection and IL-6 and CCL2/MCP-1 with pathology. Our work is pioneering the Brazilian population and corroborates data from people from other countries.

## 1. Introduction

SARS-CoV-2 was officially announced by the World Health Organization (WHO) in late 2019 as the causative agent of 2019 pandemic of coronavirus disease (COVID-19) [1,2]. COVID-19 quickly led to outbreaks of severe acute respiratory syndrome that spread across China and elsewhere in the world [2]. SARS-CoV-2 was transmitted faster and more efficiently compared to the other two epidemic coronaviruses SARS-CoV and MERS-CoV. COVID-19 threatened global public health with high human mortality and resulted in an almost complete stoppage of economic and social activities globally. Nearly 14% of patients required hospitalization, and approximately 1.4–3.4% died from COVID-19 [3,4]. On 17 February 2022, more than 416 million cases and 5,8 million deaths have been confirmed by WHO worldwide. A total of more than 10 billion doses of the vaccine have been administered [5]. The Ministry of Health of Brazil confirmed the first case on 26 February 2020 [6]. As of the end the epidemiological week 6 (end date 12 February 2022), 27,425,743 cases and 638,048 deaths were registered in Brazil [7].

The infected patient can transmit the virus through generated droplets [3]. The clinical spectrum of COVID-19 presents itself in the form of mild, moderate, or severe illness. Others have a mild influenza-like illness. Most cases present mild to moderate symptoms, characterized by fever, dry cough, sore throat, shortness of breath, and fatigue, among other symptoms. Moderate and severe cases require hospitalization and intensive care, including non-invasive and invasive ventilation, along with antipyretics, antivirals, antibiotics, and steroids [8]. Patients who developed severe forms present with severe pneumonia, acute respiratory distress syndrome (ARDS), and multiple organ failure, requiring hospitalization, intensive care, and mechanical ventilation. Men are more affected than women, and individuals with hypertension, diabetes, and obesity have worse outcomes [9].

Selection pressures lead to genetic alterations of SARS-CoV-2 and the consequent dissemination of new variants. Some of these variants have greater transmissibility, virulence, antibody evasion and reduced response to available diagnoses, vaccines, and therapy, which is why they were defined by WHO as a variant of concern (VOC) [10].

The search for antiviral and immunomodulatory therapies and effective vaccines have been carried out [11]. Several randomized clinical trials in search of potent antivirals are ongoing [12]. Some treatments have had a proven benefit, such as IL-6 receptor blockers (Tocilizumab and Sarilumab). These drugs appear to improve survival and reduce patients’ need for mechanical ventilation, despite adverse events. We also have Remdesivir that has shown antiviral activity in vitro and in vivo against SARS-CoV-2 [13,14], but there is still no evidence of its ability to improve severe cases [14].

By February 2022, billions of doses of nine different vaccines have been administrated worldwide, and several others vaccines are in pre-clinical and clinical development. Many of them with a safety and efficacy profile above 90% [5,15]. Regarding VOCs, there seems to be a decrease in neutralizing antibodies, in patients infected by previous strains and even in vaccinated individuals [16,17]. However, T cell responses appear to recognize these VOCs effectively [18,19]. The worldwide spread of VOCs raises concerns about the most vulnerable people, such as the elderly and those with pre-existing illnesses.

In the meantime, recommended strategies to reduce the spread of the disease include maintaining social distance and isolating suspected and confirmed cases. However, contrary to general guidelines, healthcare workers (HCWs) are required to provide direct care to patients with COVID-19 in primary and emergency care units and hospitals [20]. HCWs were severely affected during the 2003 outbreak of SARS-CoV-1 in Taiwan [21]. High frequencies of infection and deaths were reported among HCWs in the UK, the US, and China [20,22]. A similar scenario was observed in Brazil, and according to the Federal Nursing Council, on 9 February 2022, 61,726 cases and 872 deaths were reported [23].

Bandyopadhyay et al. performed a systematic review to estimate illnesses and deaths from COVID-19 in HCWs from a global perspective during the early stages of the pandemic on 8 May 2020, where data were available. A total of 152,888 HCWs had COVID-19, which corresponded to 3.9% of the total number of 3,912,156 patients with COVID-19 worldwide. The total number of reported HCWs deaths was 1413, or about 0.5% of the total number of 270,426 deaths from COVID-19 worldwide. As of 8 May 2020, 67 countries reported at least one death from HCWs related to COVID-19. Thus, fatalities and deaths from COVID-19 among HCWs occurred around the world [24].

This issue at COVID-19 between “viral loads/cytokine storm/disease severity” is not yet clear. It is challenging to find therapeutic drugs that inhibit viral replication and cytokine storms yet maintain an effective anti-viral immune response. A study by Meng L et al. identified that a high viral load does not necessarily imply a low immune response [25].

During the pandemic, we recruited HCWs and COVID-19 patients. We believe that the recruitment of these groups was a great opportunity, as there are many reports in the literature of patients but few in HCWs, especially in asymptomatic ones. Even bearing in mind that symptomatic HCWs are patients, they still constitute more exposure to infection, both in terms of the amount of virus and time of exposure compared to the general population. Our work is pioneering the Brazilian population and corroborates data from people from other countries [26]. Here, we propose to evaluate the anti-SARS-CoV-2 antibodies generated in these individuals, as well as their inflammatory response.

## 2. Materials and Methods

### 2.1. Study Design

Between March and June 2020, 134 individuals, 97 HCWs and 37 COVID-19 patients over 18 years of age were recruited from Rede–Casa Hospitals, Rio de Janeiro, Brazil. All answered a questionnaire with information related to COVID-19, along with histories of diseases and vaccines. Written informed consent was obtained from participants prior to any procedure. SARS-CoV-2 infection was confirmed by real-time quantitative reverse transcription polymerase chain reaction (RT-PCR) and/or serology. Case definitions of confirmed COVID-19 patients were according to the Ministry of Health of Brazil, 2020 [27]. HCWs and COVID-19 patients were stratified: HCWs who did not report having any symptoms at the time of collection (asymptomatic HCWs), those who presented acute symptoms (symptomatic HCWs or COVID-19 patients), those who died (dead patients) or those who had no more symptoms at the time of collection (recovered patient). To assess whether asymptomatic individuals remained asymptomatic and symptomatic individuals did not have persistent clinical manifestations, we followed them by telephone for four months from the date of blood draw. Plasma from 32 healthy non-COVID-19 donors was obtained in the years prior to the pandemic or even during the pandemic as control.

### 2.2. Ethics Statement

The study was conducted according to the guidelines of the Declaration of Helsinki. All data were treated confidentially and anonymously in compliance with the National Commission for Research Ethics (number 57221416.0.1001.5248. version 6), Ethics Research Committee of Hospital Casa Rio-Botafogo (number 47885515.8.0000.5279). Written informed consent was obtained from all participants.

### 2.3. Blood Sample Collection

After participants had given informed consent, blood was collected via venipuncture using BD Vacutainer™ tubes containing acid-citrate-dextrose (Cat. # BD 364606). Approximately 10 mL of peripheral blood was achieved, and plasma was obtained by centrifugation. Different aliquots of plasma were stored at −70 °C until analysis.

### 2.4. SARS-CoV-2 Serology

SARS-CoV-2 S1 protein-specific binding IgA and IgG were analyzed by enzyme-linked immunosorbent assay (ELISA) according to the manufacturer’s instructions (Cat. #EI 2606-9601 A and #EI 2606-9601 G, Euroimmun, Lübeck, Germany).

### 2.5. The Plaque Reduction Neutralization Test (PRNT)

As previously described, a 50% plaque reduction neutralizing titer (PRNT50) was used to investigated neutralizing antibodies [28]. Briefly, plasma samples were heat-inactivated at 56 °C for 30 min and then tested for their ability to neutralize plaque-forming units (PFU) of the SARS-CoV-2 B.1 strain isolated from a patient in Rio de Janeiro, Brazil. In a biosafety level 3 platform, inactivated plasma aliquots were initially tested in 1:10 dilutions. Those that neutralized SARS-CoV-2 by at least 50% were tested in serial two-fold dilutions to confirm and determine the final 50% endpoint titers. Plasma samples were kept frozen at −20 °C until use. All samples with PRNT titers equal to or greater than 10 in the screening assay were retested in endpoint assays from 1:10 to 1:320 for endpoint titers. Samples were considered seropositive for SARS-CoV-2 neutralizing antibodies when at a dilution of at least 1:10 it was able to reduce 50% or more of SARS-CoV-2 PFU following standard protocols [29].

### 2.6. Quantification of Cytokines and Chemokines

Plasma cytokine and chemokine levels of the following six molecules were measured using ELISA kits, in accordance with the manufacturer’s instructions: interferon gamma (IFN-γ) (cat. 900-K27, Peprotech, New Jersey, NJ, USA); tumor necrosis factor alpha (TNF-α) (cat. 900-K25, Peprotech); C-C motif chemokine ligand 2 (CCL2/ MCP-1) (cat. 900-K31, Peprotech); C-X-C motif chemokine ligand 10/ interferon protein-10 (CXCL10/IP-10) (cat. 900-K39, Peprotech); interleukin-6 (IL-6) (cat. DY206, R&D Systems, Minneapolis, MN, USA); and IL-10 (cat. DY217B, R&D Systems). Standard curves of known concentrations of recombinant human cytokines or chemokines were used to convert optical density (OD) into concentration units (pg/mL). The plates were read in a SpectraMax Paradigm^®^ machine (Molecular Devices, CA, USA).

### 2.7. Quantification of IFNs

Plasma samples from non-COVID-19 donors and COVID-19 patients were analyzed using a multiplex Human type 1/2/3 Interferon panel (cat. 740396, LEGENDplexTM Human Anti-Virus Response Panel, BioLegend, San Diego, CA, USA) containing the following IFNs type 1 (IFN-α, IFN-β), 2 (IFN-γ), and 3 (IFN-λ1 and, IFNs-λ2/3). The samples were analyzed on a CytoFLEX Flow Cytometer (Beckman Coulter, Indianapolis, IN, USA) according to instructions from the manufacturer.

### 2.8. Statistical Analysis

Antibody levels and cytokine/chemokine concentrations between groups were calculated using the Kruskal–Wallis test followed by Dunn’s multiple comparisons test. Differences in seropositivity rates were assessed using Fisher’s exact test. A correlation analysis was performed using a nonparametric Spearman test. All statistical and graphical analyzes were performed using GraphPad Prism 6.0 software (San Diego, CA, USA). Values of *p* < 0.05 were considered statistically significant.

Principal component analysis (PCA) between asymptomatic and symptomatic patients was used to reduce dimensionality and identify patterns of cytokines and chemokines associated with disease severity. The Kaiser–Meyer–Olkin (KMO) and Bartlett sphericity tests were used to assess whether the data were adequate for factor analysis. PCA with subsequent varimax rotation determined the number of principal components (eigenvalues > 1.0) and the cumulative percentage of variance. The individual scores for each element were transformed into a scale from 0 to 1 and the difference between the groups of patients evaluated.

The area under the receiver operating characteristics (ROC) curve (AUC) was estimated to discriminate severity. The optimal cutoff value was defined as the value with the highest Youden index. The R Statistical Language Version R-4.04 for Windows [30].Values of *p* < 0.05 were considered statistically significant.

## 3. Results

### 3.1. Baseline Characteristics of the Study Population

The demographic and clinical characteristics of the 134 participants are shown in Table 1. The median age of healthy non-COVID-19 donors was 34 (22–62), and 59% (19/32) were women. Donors showed no signs of infection, fever or malaise, or any chronic illness.

Ninety-seven HCWs were registered, of which 45% (44/97) were classified as asymptomatic, as they did not report symptoms at the time of blood collection. The median age of asymptomatic HCWs was 37 (29–42), and 73% (32/44) were women. Among acutely symptomatic HCWs, the median age was 34 (27–41), and 47% (7/15) were women. The majority of symptomatic HCWs enrolled had mild symptoms. Common symptoms were cough (53%), headache (47%), and diarrhea (40%). The median post-symptom onset (PSO) was 7 (range 4–12).

Regarding patients with COVID-19, the median age of symptomatic patients was 61 (48–69), and 60% (9/15) were women. In addition, the average PSO was 12 (4–19). Symptomatic patients were classified according to disease severity as mild/moderate (individuals with signs and symptoms of COVID-19 who had oxygen saturation ≥ 94%), severe (patients admitted to the Intensive Care Unit (ICU) with saturation oxygen <94%), and who died of COVID-19.

Symptomatic patients with COVID-19 had a cough (60%), fever (47%), and dyspnea (40%). The majority (80%; 12/15) of symptomatic cases of COVID-19 were hospitalized and treated in the ICU. These patients were discharged alive. Eight serious problems died during hospitalization. The mean age of patients who died was 76 (71–85), and 62% (5/8) were women. On admission to the hospital, the most frequent symptoms were dyspnea (100%), cough, and fever (63%). All fatal cases required mechanical ventilation. Fatal cases had a high Charlson comorbidity rate [4 (3–5)], reflecting a significant comorbid burden such as hypertension (50%) and diabetes mellitus (25%). Co-infection with the influenza A (H1N1) virus was detected in 3 of 8 fatal cases (37%).

We also included in the study a group of 14 patients with COVID-19 and 38 HCWs who recovered from COVID-19. Concerning recovered patients, the median age was 36 (29–47), and 73% (38/52) were women. The most common acute symptoms reported by recovered participants were headache, cough, and runny nose (63%), fatigue (52%), and myalgia (50%). Two participants from the recovered group were hospitalized. They were discharged without complications from the disease. Three recovered patients belonged to people living with HIV (PLHIV) and on highly active antiretroviral therapy (HAART). None of the PLHIV were hospitalized. Among comorbidities, hypertension (47%) was the most common among them, followed by obesity (6%) and diabetes (2%).

Most symptomatic patients and those who died underwent molecular diagnosis, and, among them, 20 (20/23; 86%) were positive. For patients who died, seven (87.5%) were RT-PCR positive. Only three recovered patients (3/14; 21%) performed molecular diagnosis in the acute phase of the disease, and all were RT-PCR positive.

### 3.2. Anti-SARS-CoV-2 IgA and IgG Antibody Responses in Healthcare Workers and Patients Exposed or Even Infected to SARS-CoV-2 during the Pandemic

**Figure 1:**
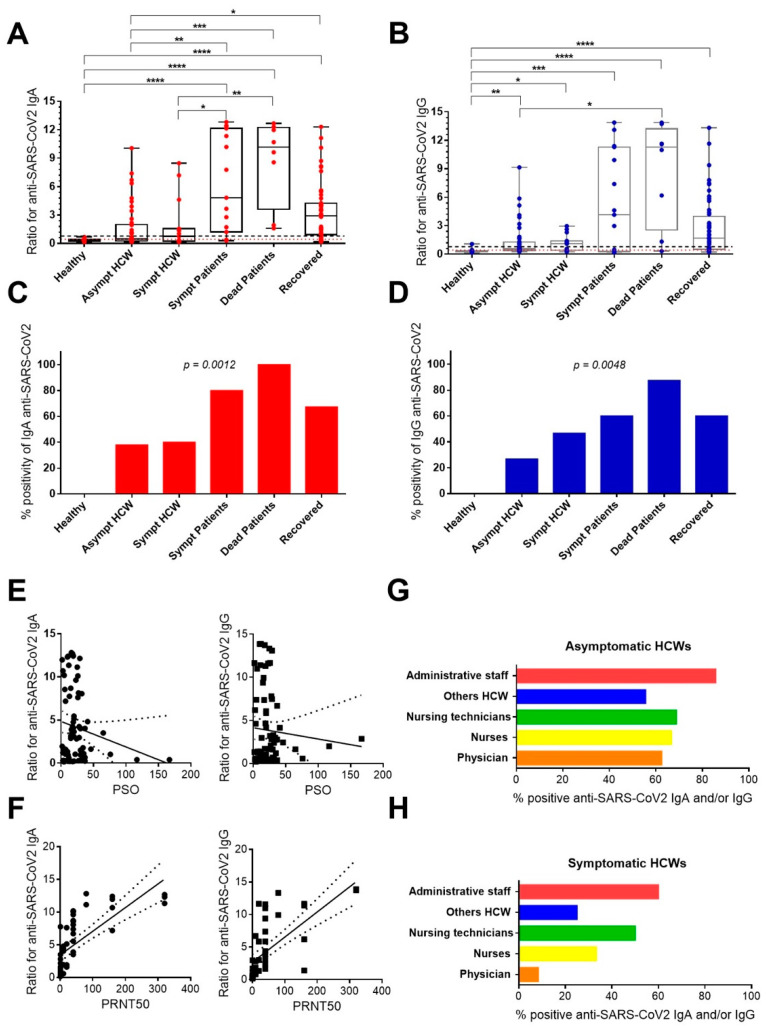
IgA and IgG seropositivity in HCWs and COVID-19 Patients exposed and/or infected to SARS-CoV-2 during the pandemic. According to the manufacturer’s instructions, the results are expressed by calculating the ratio of the absorbance value of the control or patient sample to the absorbance value of the calibrator. (**A**) Comparison of specific IgA and (**B**) IgG ratios in non-COVID-19 healthy individuals (n =  17), asymptomatic HCWs (n =  44), acute symptomatic HCWs (n = 15), acute symptomatic COVID-19 patients (n = 15), COVID-19 patients who died (n = 8) and patients recovered from COVID-19 (n = 52). The box plots show the median (middle line), and the whiskers show minimum to maximum quartiles above and below the box. Dashed black line represents cutoff for positivity (ratio ≥ 1.1). Dotted red line corresponds to cutoff for negativity (ratio < 0.8). The *p* values were calculated using the Kruskal–Wallis test followed by Dunn’s multiple comparisons test. (**C**) Changes to seropositivity rates for specific IgA and (**D**) IgG among groups. (**E**) Spearman correlation test was applied on the data means of PRNT_50_ for detection of SARS-CoV-2-neutralizing antibodies with the days of post-symptoms onset (PSO) and (**F**) with anti-SARS-CoV-2 IgA and IgG ratios. (**G**) Percentage of seropositivity (IgA and/or IgG) among asymptomatic HCWs physician (n  =  12), nurses (n  =  6), nursing technicians (n  =  12), other HCWs (n  =  4) and administrative staff (n  =  10). (**H**) The same analysis for symptomatic HCWs (acute and recovered) physicians (n  =  8), nurses (n  =  6), nursing technicians (n  =  16), other HCWs (n  =  9) and administrative staff (n  =  7). The columns in (**C**,**D**) to (**G**,**H**) show the means. Statistical significance for differences in the positivity frequency was assessed using Fisher’s exact test. Asterisks indicate significant differences (* *p* < 0.05; ** *p* < 0.01; *** *p* < 0.001; **** *p* < 0.0001).

The overall plasma IgA and IgG profile against SARS-CoV-2 in a total of 134 samples obtained from 97 HCWs and 37 patients with COVID-19 are shown in Figure 1.

According to the manufacturer’s instructions, the results are expressed by calculating the ratio of the absorbance value of the control or patient sample to the absorbance value of the calibrator.

All samples from the control group produced negative results, as the mean anti-SARS-CoV-2 plasma IgA and IgG ratios were 0.303 (IQR, 0.212–0.433) and 0.272 (IQR, 0.245–0.355), respectively.

Asymptomatic HCWs had lower median antibody ratios than all COVID-19 patient groups. Between asymptomatic or symptomatic HCWs and healthy donors no statistical differences were found. The difference in anti-SARS-CoV-2 IgA ratios between symptomatic HCWs and patients with symptomatic COVID-19 or deceased patients was statistically significant (Figure 1A).

Anti-SARS-CoV-2 IgG was more detected in HCWs and all COVID-19 patient groups compared to healthy donors. Anti-SARS-CoV-2 IgG did not discriminate between the groups of HCWs and COVID-19, except between asymptomatic HCWs and deceased patients (Figure 1B).

Considering the detection values above the positive control established through the commercial test (ratio ≥ 1.1), the number of positive samples for specific IgA for SARS-CoV-2 was 52 out of 97 (53.6%) HCWs and 26 out of 37 (70.3%) in patients with COVID-19. For SARS-CoV-2, the specific IgG was 43 out of 97 (44.3%) HCWs and 23 out of 37 (62.2%) patients with COVID-19. As shown, the frequencies of positivity in anti-SARS-CoV-2 IgA and IgG differentiated between HCW and evaluated patients (Figure 1C,D).

A positive correlation was found between the detection of anti-SARS-CoV-2 IgA and IgG among all HCWs and COVID-19 patients (r = +0.7438; *p* < 0.0001), only in HCWs (r = +0.7137; *p* < 0.0001) and only in COVID-19 patients (r = +0.8345; *p* < 0.0001). However, no correlation was found between the number of PSO with anti-SARS-CoV-2 IgA or IgG (Figure 1E).

None of the samples in the control group had PRNT50 titers for SARS-CoV2. However, plasma samples from 15 of 19 (78.9%) HCWs and 16 of 20 (80%) patients with COVID-19 had PRNT50 titers ranging from 10 to 320. A strong positive correlation was found between the titers of PRNT50 with measures of anti-SARS-CoV-2 IgA (r = +0.8671; *p* < 0.0001) and anti-SARS-CoV-2 IgG (r = +0.8314; *p* < 0.005) among all HCWs and patients (Figure 1F).

Finally, we investigated which HCWs were most exposed to SARS-CoV-2. In ascending order of positivity, asymptomatic HCWs in the administrative staff with 60% (6/10), followed by 50% (6/12) nursing technicians, 33.3% (2/6) of nurses, 25% (1/4) of the other HCWs, and 8.3% (1/12) of the physicians had specific IgA and/or IgG for SARS-CoV-2. Statistical analyzes did not indicate significant differences between the five subgroups (Figure 1G). For symptomatic HCWs (acute and recovered), the administrative staff appears first with 85.7% (6/7), followed by 68.8% (11/16) of nursing technicians, 66.7% (4/6) of nurses, 62.5% (5/8) of physicians, and 55.6% (5/9) of other HCWs had IgA and/or IgG specific for SARS-CoV-2. No differences were observed between the five subgroups (Figure 1H).

### 3.3. Detection of IFN-λ2/3, IFN-γ and TNF-α in HCWs and COVID-19 Patients in Whom Anti-SARS-CoV-2 Antibodies Had Been Detected

Only individuals with detection of IgA and/or IgG were analyzed to quantify plasma inflammatory mediators. As a control, the physiological levels of inflammatory mediators used the healthy seronegative donor group to control the physiological levels of inflammatory mediators.

**Figure 2:**
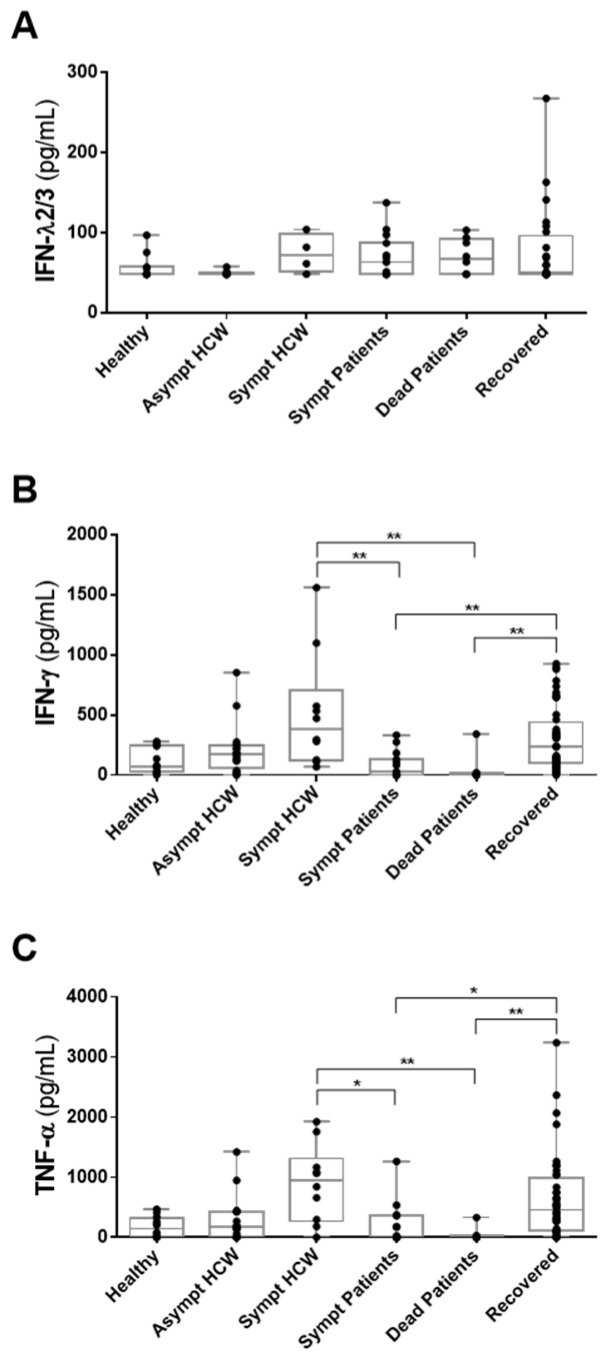
Comparison of IFN-λ2/3, IFN-γ and TNF-α concentrations in different groups of HCWs and COVID-19 patients who have detectable anti-SARS-CoV-2 antibodies. Plasma samples from non-COVID-19 healthy individuals (n  =  11–13), asymptomatic HCWs (n  =  8–16), acute symptomatic HCWs (n = 4–10), acute symptomatic COVID-19 patients (n = 15), COVID-19 patients who died (n = 8) and patients recovered from COVID-19 (n = 24–44). (**A**) IFN-λ2/3; (**B**) IFN-γ; and (**C**) TNF-α. The box plots show the median (middle line), and the whiskers show minimum to maximum quartiles above and below the box. The *p* values were calculated using the Kruskal–Wallis test followed by Dunn’s multiple comparisons test. * *p* < 0.05; ** *p* < 0.01.

The data demonstrate that IFN-λ2/3 levels did not differentiate HCWs and COVID-19 patients from healthy donors. However, asymptomatic HCWs tended to have lower IFN-λ2/3 than acute patients or recovered patients (Figure 2A). IFN-γ and TNF-α profiles were very similar between groups. Both IFN-γ and TNF-α were significantly higher in symptomatic HCWs compared to symptomatic and deceased patients with COVID-19. Recovered individuals still maintain elevated levels of IFN-γ and TNF-α compared to symptomatic and dead patients from COVID-19 (Figure 2B,C).

### 3.4. Detection of CXCL10/IP-10, IL-6, IL-10 and CCL2/MCP-1 in HCWs and COVID-19 Patients in Whom Anti-SARS-CoV-2 Antibodies Had Been Detected

Likewise, we selected all individuals in which specific IgA and/or IgG were detected to quantify these cytokines and chemokines.

**Figure 3:**
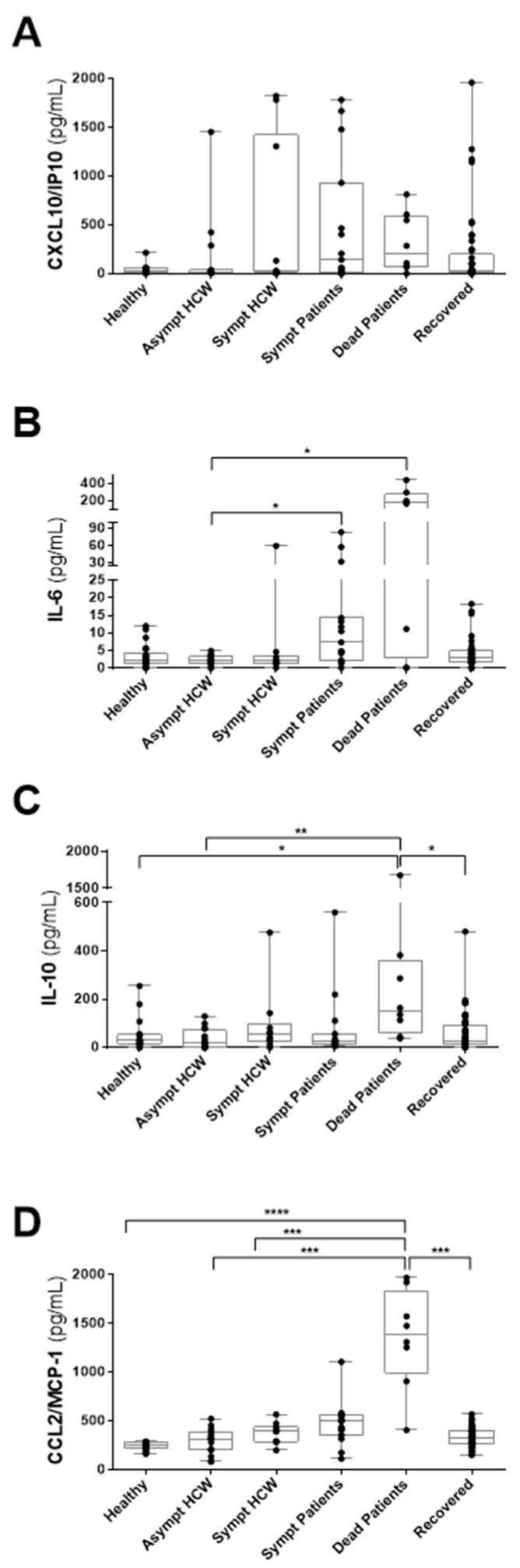
Comparison of CXCL10/IP-10, IL-6, IL-10 and CCL2/MCP-1 concentrations in different groups of HCWs and COVID-19 patients who have detectable anti-SARS-CoV-2 antibodies. Plasma samples from non-COVID-19 healthy individuals (n = 14–22), asymptomatic HCWs (n  =  15–16), acute symptomatic HCWs (n = 10), acute symptomatic COVID-19 patients (n = 15), COVID-19 patients who died (n = 8) and patients recovered from COVID-19 (n = 44–45). (**A**) CXCL10/IP-10; (**B**) IL-6; (**C**) IL-10; and (**D**) CCL2/MCP-1. The box plots show the median (middle line), and the whiskers show minimum to maximum quartiles above and below the box. The *p* values were calculated using the Kruskal–Wallis test followed by Dunn’s multiple comparisons test. * *p* < 0.05; ** *p* < 0.01; *** *p* < 0.001; **** *p* < 0.0001.

CXCL10/IP-10 measurements were not different between groups (Figure 3A). Patients who died from COVID-19 stand out for their elevated levels of IL-6, IL-10, and CCL2/MCP-1. IL-6 was detected more in dead and symptomatic patients with COVID-19 than in asymptomatic HCWs (Figure 3B). In comparison, IL-10 was significantly higher in COVID-19 patients who died than in healthy donors, asymptomatic HCWs, and recovered groups (Figure 3C). Patients with COVID-19 who died had elevated levels of CCL2/MCP-1 compared to healthy donors, asymptomatic and symptomatic HCWs, and, also, recovered individuals (Figure 3D).

### 3.5. Principal Component Analysis (PCA) of Cytokines/Chemokines According Asymptomatic and Illness Severity

Principal component analysis (PCA) is an unsupervised dimensionality reduction method, suitable for analyzing multiple biological variables that present interdependence, removing noise, but retaining information of interest for the understanding of hidden structures and patterns. PCA is ideal for exploring multiple inflammatory mediators whose main characteristics are pleiotropism, redundancy, synergism, or antagonism [31].

**Figure 4:**
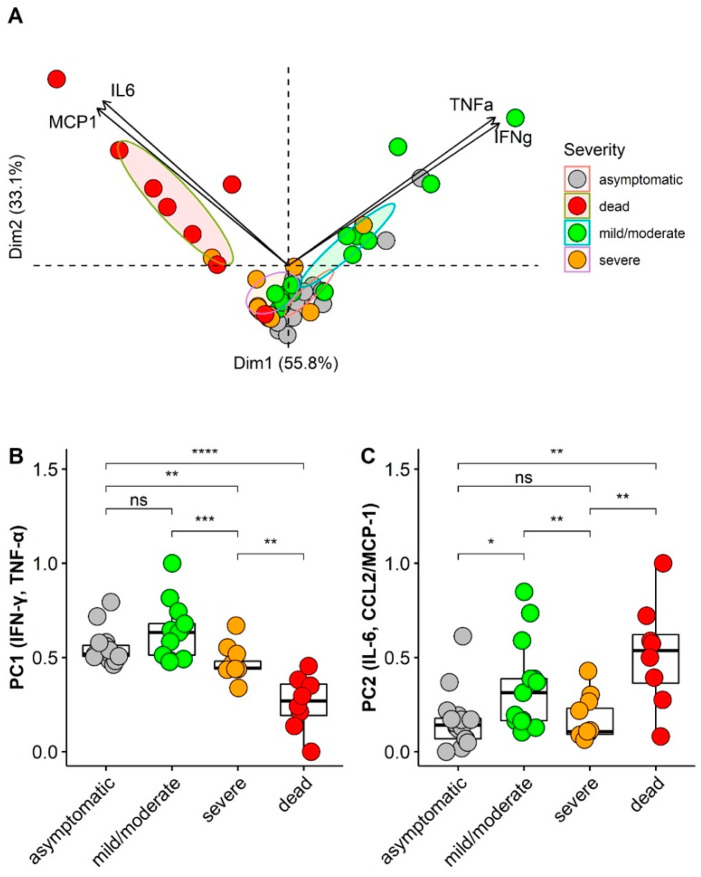
Cytokines/Chemokines profiles in SARS-CoV-2 infected patients. (**A**) PCA biplot shows the two principal components that represent a cumulative percentage of variance of 88.9%, PC1 (55.8%) and PC2 (33.1%). Individual scores for each PC are represented as colored points that are grouped according to severity (ellipses represent 95% confidence interval for each group). Loadings of variables are represented as arrows; the length of each arrow represents the contribution of each variable on each principal component; (**B**) Comparison of individual scores for PC1 (IFN-γ and TNF-α); (**C**) Comparison of individual scores for PC2 (IL-6 and CCL2/MCP-1); * *p* < 0.05; ** *p* < 0.01; *** *p* < 0.001; **** *p* < 0.0001.

We performed PCA analysis for cytokines and chemokines from 49 asymptomatic HCWs and symptomatic individuals, including HCWs and patients. The analysis identified two different cytokine/chemokine combinations (eigenvalue criteria > 1.0) with a cumulative percentage of the variance of 88.9% after varimax rotation. The KMO test value was 0.54, and the Bartlett test was significant (*p* < 0.001). The proinflammatory cytokines IFN-γ and TNF-α contributed mainly to the first major component (PC1). In contrast, the proinflammatory cytokine IL-6 and the chemokine CCL2/MCP-1 had an orthogonal contribution to the second major component (PC2) (Figure 4A). For PC1, higher individual score values were observed among asymptomatic HCWs and mild/moderate patients than among severe and dead patients (*p* < 0.001). For PC2, the deceased patients had higher personal score values than the other groups (*p* = 0.002) (Figure 4B). On the other hand, lower PC1 scores were observed in individuals aged ≥ 60 years compared to those under 60 years (*p* < 0.001), but no difference was observed between these age groups for PC2.

### 3.6. ROC Curve Analysis of Cytokines/Chemokines According Asymptomatic and Illness Severity

To identify biomarkers that could explain an increased risk of developing severe disease, we investigated the relationship between cytokine/chemokine levels and severity, using ROC analysis of 49 asymptomatic HCWs and symptomatic individuals. Like the PCA analysis, symptomatic groups were separated into mild/moderate, severe, and dead individuals. The cytokines/chemokines that demonstrated the largest areas under the curve (AUC) were IL-6 (AUC = 0.805; 95% CI 0.652–0.958) and CCL2/MCP-1 (AUC = 0.837; 95% CI 0.716–0.958) (Table 2).

**Figure 5:**
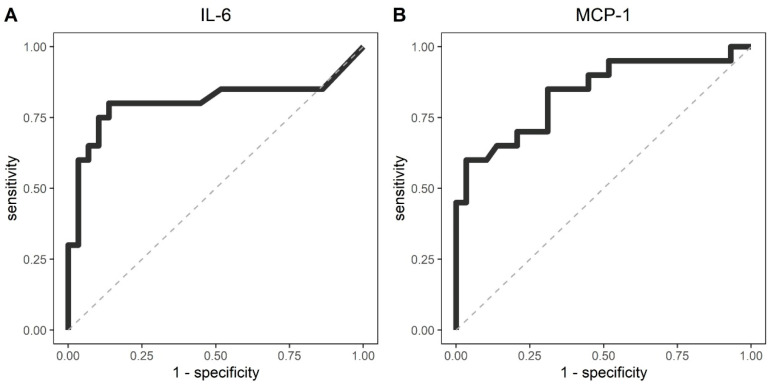
ROC analyses of cytokines and chemokines to discriminate severe cases. (**A**) ROC curve for IL-6, AUC = 0.805 (95% CI 0.652–0.958) and optimal cut-off 4.2 pg/mL (sensitivity: 80%, specificity: 86%); (**B**) ROC curve for CCL2/MCP-1, AUC = 0.837 (95% CI 0.716–0.958) and optimal cut-off was 547.8 pg/mL (sensitivity: 60%, specificity: 96%).

The optimal calculated cutoff values for IL-6 and CCL2/MCP-1 to predict severity were 4.2 pg/mL (sensitivity: 80% and specificity: 86%; positive predictive value: 80%, negative predictive value: 86%) and 547.8 pg/mL (sensitivity: 60% and specificity: 96%; positive predictive value: 92%, negative predictive value: 78%), respectively (Figure 5).

## 4. Discussion

During the pandemic, we recruited healthcare workers (HCWs) and COVID-19 patients. Our hypothesis is that the specific humoral response associated with neutralizing activity and the regulated production of immunological mediators are characteristics of individuals with better clinical evolution or recovered. Additionally, asymptomatic HCWs and non-critical patients with COVID-19 produce “sufficient” amounts of inflammatory mediators, inducing viral clearance and protection. In contrast, symptomatic HCWs and critically ill patients with COVID-19 have “insufficient” amounts of antiviral cytokines but “exaggerated” quantities of inflammatory cytokines, leading to pathologies. It is important to remember that the same mediators play a fundamental role in controlling the pathogen and restoring homeostasis. Through different study groups, it was possible to compare the levels of these mediators.

An efficient antiviral immune response state is mediated by antibodies and the production of inflammatory mediators, cytokines, and chemokines, constituting some of the various arms of immunity. On the other hand, high anti-SARS-CoV-2 antibodies have already been associated with the development of multisystem inflammatory disease, and cytokine storms are strongly associated with ARDS and multiple organ failure [32]. Antibody response to SARS-CoV-2 can be detected during infection or vaccination. However, several outstanding issues remain to be addressed regarding the magnitude and persistence of antibody titer and its correlation with the strength of the immune response.

The main finding of our study was that detectable levels of IgA and IgG were observed in HCWs who declared themselves asymptomatic. Some concern shows that a considerable proportion of asymptomatic HCWs (who would therefore not be forced to stay at home) was, therefore, potentially a means of transmission to their families and their working environment [33].

Regarding the clinical evolution, Li et al. reported that most of the infected medical staff had milder symptoms and a better prognosis than hospitalized patients [34]. Onchonga et al. reported a 60.4% increase in self-medication primarily by nurses due to migraine and headaches during the COVID-19 outbreak [35]. Other studies have reported headache attacks during the pandemic [36] and after vaccination [37]. Exploring univariate regression analysis, Trigo et al. found a lower probability of worse prognosis or death among headache patients [38]. On the other hand, the headache was more frequently present in deceased cases than survivors [39]. We found increased headache frequency in symptomatic healthcare professionals and recovered COVID-19 patients; however, we must interpret these findings with caution.

A recent study showed similar viral loads in symptomatic patients compared to asymptomatic individuals, highlighting the potential for transmission of SARS-CoV-2 carriers despite their clinical status [40]. In contrast, Gao et al. suggested potentially low infectivity in asymptomatic carriers of SARS-CoV-2, as none of the 455 exposed contacts tested positive [41]. These results agree with the studies carried out by Canova et al. in which none of the exposed HCWs had a subsequent positive SARS-CoV-2 test [42]. All asymptomatic individuals investigated in the study by Gao et al. were wearing a mask, further reducing the spread of infection [41]. However, this low infectivity, the potential for silent transmission still represents a major problem that needs to be addressed, especially in low- and middle-income countries without medical resources.

Considering that almost half of the RT-PCR positive HCWs were asymptomatic, there is an urgent need to promote a process of continuity, systematic screening of all HCWs in high-risk settings, use of adequate PPE, and other standardized procedures. In addition, a low threshold for suspicion of infection in low-risk patient settings is needed to promote early isolation to avoid cross-infection [43].

The detection of specific IgM and IgG for SARS-CoV-2 has been performed by most studies, but few have detected specific IgA for SARS-CoV-2 [44].Therefore, Ma H et al. evaluated a total of 87 confirmed COVID-19 patients and used a set of chemical luminescence kits to detect the presence of receptor-binding domain (RBD) specific IgA, IgM, and IgG. According to the authors’ data, IgA detection showed greater sensitivity between 4 to 25 days after disease onset, peaking at 16–20 days followed by a decline, but remaining relatively high up to 31–41 days. Specific IgG was lowest in the early stages of the disease, but increased 15 days after disease onset, peaked at 21–25 days, and remained at a relatively high reading for up to 31–41 days. IgM peaked in the early stages, but the lesson was lower than that of IgA or IgG. Furthermore, the authors found that IgA levels in severe cases were significantly higher than in mild or moderate cases. In comparison, serum IgM and IgG levels in moderate and severe patients were substantially higher than in mild cases. No difference was found between severe and moderate [45].

A higher frequency of specific antibodies was observed among administrative employees and nursing technicians, which can be explained by greater exposure to the virus, as they usually use public transport. Traveling by public transport is considered dangerous as SARS-CoV-2 is a highly contagious pathogen, with many asymptomatic individuals [46]. A study in Brazil confirmed that people who use public transport are more exposed to contracting the coronavirus [47].

More research is needed to examine whether high antibody levels indicate exposure to a high viral load and whether these levels confer long-term immunity. Peirlinck et al., using a mathematical model, demonstrated that the differences in the transmission of symptomatic and asymptomatic individuals are exclusive during the infectious periods [48]. Similar models support the role of asymptomatic carriers in the rapid spread of the disease [49] in China [50] and Italy [51]. The ability of asymptomatic and pre-symptomatic people to transmit the virus before symptom onset is well documented [52,53]. There is a consensus that infection rates are higher among household and family contacts (~5%) than among health center contacts (0.9%) [54].

A dysregulated host immune response becomes ineffective as it is associated with severe pulmonary pathology, particularly in patients infected with SARS-CoV2 who have died. There is an overactivation of immune cells and their signaling molecules, leading to the “cytokine storm.” Among patients infected with SARS-CoV2, the “cytokine storm” is often associated with a “flood” of immune cells and their products in the lung, a phenomenon also observed in SARS-CoV [55,56] and MERS-CoV [57,58,59].

There have been many advances in the knowledge of the pathophysiology of COVID-19. However, the association between viral load and disease severity in patients with COVID-19 is still controversial [60]. Furthermore, it is challenging to find therapeutic drugs that inhibit viral replication and inhibit the accompanying cytokine storm and maintain an appropriate immune response. A study by Meng L et al. identified genes differentially expressed in cells infected with SARS-CoV-2 and uninfected cells and the relationship with disease severity. The authors demonstrated that epithelial cells had a low viral transcriptional load, whereas primary immune cells had high infection rates. A high viral load in epithelial cells was not implicated in a severe ARDS outcome or poor recovery. On the other hand, gene signatures related to increased viral load fall into the mild or moderate group [25].

Collectively, our data suggest that symptomatic HCWs had an increased antiviral response with high concentrations of IFN-γ and TNF-α, but an equal balance of inflammatory molecules such as IL-6, IL-10, and CCL2/MCP-1 to healthy individuals. The question that arises is whether this is the best image of an effective anti-SARS-CoV-2 immune response, or is it just a reflection of the stress experienced by these professionals? Few studies address the issue of stress and immunological changes with HCWs, especially in the pandemic period. The article published by Jung YH et al., talks a little about the subject. Although this study addressed the general population, it demonstrated some very interesting correlations. Briefly, the group investigated different associations, including cytokines and stress in 70 healthy individuals. High IL-10 levels were significantly associated with low-stress levels only in the right brain dominant group. The cytokines TNF-α and IL-6 had negative effects, while the cytokines IL-10 and IFN-γ showed beneficial effects on stress levels and multiple dimensions of emotional and cognitive intelligence. Thus, cytokines, stress, and emotional and mental intelligence are closely linked [61]. Therefore, changes observed in HCWs may reflect the stress experienced by these people. We are following these HCWs to assess these measures after vaccination.

Our data suggest an impairment of the antiviral inflammatory response mediated by IFN-γ and TNF-α in patients who died from COVID-19. Intense inflammation reflected by the increase in IL-6, IL-10, and CCL2/MCP-1 may have contributed to the worsening of the disease. PCA confirmed these data and allowed us to identify the profiles of two cytokines/chemokines in patients with COVID-19. The first possible protection profile is significantly elevated levels of IFN-γ and TNF-α in asymptomatic HCWs and patients with mild/moderate disease. The second pathological profile is characterized by elevated IL-6 and CCL2/MCP-1 in critically ill and deceased patients. These findings are consistent with a recently published study that showed high but non-significant levels of IFN-α, IFN-γ, and TNF-α in asymptomatic individuals and mild cases and a significant increase in IL-6 in patients with a severe and critical illness [62]. Previous studies have established that elevated IL-6 levels are predictive criteria for COVID-19-associated cytokine storm [63]. The use of glucocorticoids such as dexamethasone can reduce local and systemic IL-6 production and mitigate COVID-19-associated respiratory distress syndrome [64].

Among the many manuscripts that discuss IL-6 levels related to SARS-CoV-2 infection, a recent study by Wu J et al. in 1472 hospitalized patients with COVID-19 made a very interesting analysis. The authors demonstrated that, indeed, an understanding of the kinetics of IL-6 levels is essential for monitoring and treating patients infected with SARS-CoV-2. The results indicated that IL-6 was closely related to age, sex, body temperature, blood oxygen saturation (SpO2), and underlying disease. As a stable indicator, changes in IL-6 levels can indicate inflammatory conditions during a viral infection. Two specific treatments, namely tocilizumab and convalescent plasma therapy, lowered IL-6 and alleviated inflammation. They also found that patients with IL-6 levels, which were 30 times higher than normal, had a poor prognosis compared to patients with lower IL-6 levels [65].

Our findings from the ROC curve analysis support that IL-6 and CCL2/MCP-1 can be predictive markers of severity. Although our calculated cutoff value was lower for IL-6 and higher for CCL2/MCP-1, this could be due to differences in measurement techniques and definition of clinical parameters.

Standardized prospective studies with a larger sample size will be needed to validate these cutoff values. Hospitalized patients with COVID-19 who did not require ventilatory support had high levels of type I interferon (IFN-α and IFN-β). In contrast, patients who required mechanical ventilatory support had increased IL-6, IL-8, TNF -α, and IL-10 [66]. As is well known, after viral replication, the immune response is initiated by innate defenses, mediated by pattern recognition receptors (PRRs). Subsequently, the activation of transcription factors such as interferon regulatory factors (IRFs) and nuclear factor-kB (NF-kB) induces the production of cytokines, chemokines, and type I, II, and III interferons (IFN) [67]. An elevated expression of interferon-stimulated cytokines and genes (ISGs) in the lung suggests that SARS-CoV-2 elicited a robust IFN response [68]. However, studies have shown that the virus effectively prevents innate immune responses associated with IFN types I and III in vitro [69] and patients [70]. In our study, HCWs and patients with COVID-19 had no change in plasma IFN-λ2 / 3 levels compared to the healthy group. However, further investigation, including more acute samples, will be needed.

There are some limitations to this study. We used 134 plasma samples from only 20 patients confirmed with COVID-19 through molecular testing, and plasma samples were not available every day for all patients. The most recent plasma was collected on the 2nd day and the last on the 167th day after the onset of self-reported disease. Only 28 cases of serum samples were collected in the first 15 days after disease onset, which consequently influenced accuracy. Furthermore, only 49 individuals can analyze the PCA and ROC curves. However, this study provides valuable information about the serological responses of patients with HCWs and COVID-19, especially concerning inflammatory mediators.

## 5. Conclusions

This study brings some news and contributions to a better understanding of the immunopathogenesis of COVID-19 in the Brazilian population. The analysis with five different groups, including HCWs and COVID-19 patients, confirmed some known associations, such as old age, diabetes, and hypertension as risk factors for COVID-19. Age has always been seen as a factor associated with the severity (or not) of COVID-19. Elderly HCWs were laid off during the pandemic; Younger patients are more resistant, while the elderly is more susceptible. The age variation is certainly a bias in our study. However, when we carried out fieldwork, going to hospitals to recruit HCWs and patients, the real scenario consisted of this population presented in the study. However, we point out headaches as a frequent symptom in symptomatic HCWs, which may also be related to the stress experienced by these workers. The role of administrative staff and nursing technicians who seem to have been the most affected by this pandemic may be because of their living conditions. Also, a few reports in the literature address IgA. Here we con-tribute to indicate IgA as a potential biomarker of gravity since optical density and % positivity, together with IgG, were higher in dead COVID-19 cases. The permanence of these antibodies in recovered patients after a long while until the acute infection was surprising. Furthermore, we confirmed a strong direct correlation between antibody detection and neutralizing activity [71,72].

Finally, we highlight the balance of an almost physiological response of all mediators studied in asymptomatic HCWs, very similar to healthy donors. We detected the highest levels of IFN-γ and TNF-α in symptomatic HCWs and recovering patients. We still don’t know how to explain these data, but it is likely that, due to constant exposure to the virus by symptomatic HCWs, it could somehow restimulate the immune cells that produce these cytokines, maintaining an antiviral alert status in these workers. High levels of IFN-γ and TNF-α in recovered patients compared to sick patients could explain a good resolution of the disease in this group, which did not occur in severe cases and deaths. Either way, they are speculations, bringing more possibilities for further in-depth future investigation. Finally, elevated IL-6, IL-10, and CCL2/MCP-1, especially in severe cases and deaths, probably contribute to the “cytokine storm.” Therefore, based on our data and all the existing literature, we can suggest biomarkers of good versus bad resolution of the disease. Thus, host immunity needs to be regulated through an increased antiviral response associated with modulation of the pro-inflammatory response.

## Figures and Tables

**Table 1:** Demographic and clinical characteristics of Health care workers (HCWs) and COVID-19 patients.

	Asymptomatic HCWs	Symptomatic HCWs	Symptomatic Patients	Dead Patients	Recovered HCWs/Patients	*p* *
Total	44	15	15	8	52	
Gender, n (%)						
Female	32 (73)	7 (47)	9 (60)	5 (62.5)	38 (73)	0.303
Age (years) ^a^	37 (29–42)	34 (27–41)	61 (48–69)	76 (71–85)	36 (29–47)	<0.001
Post- symptoms onset	NA	7 (4–12)	12 (4–19)	7 (3–14) ^c^	27 (19–33)	<0.001
Signs/Symptoms, n (%)						
cough ^b^	0	8 (53)	9 (60)	5 (63)	33 (63) ^c^	0.956
headache	0	7 (47)	3 (20)	1 (13)	33 (63)	**0.003**
coryza	0	4 (27)	2 (13)	1 (13)	33 (63)	**<0.001**
fatigue	0	4 (27)	5 (33)	3 (38)	27 (52)	0.286
myalgia	0	4 (27)	4 (27)	3 (38)	26 (50)	0.234
fever	0	4 (27)	7 (47)	5 (63)	21 (40)	0.420
dyspnea	0	2 (13)	6 (40)	8 (100)	20 (38)	**0.001**
diarrhea	0	6 (40)	3 (20)	0	15 (29)	0.208
Hospitalization, n (%)	0	0	12 (80)	8 (100)	2 (4)	**<0.001**
Comorbidity, n (%)						
hypertension	0	1 (7)	7 (47)	4 (50)	7 (47)	**<0.001**
diabetes	0	0	5 (33)	2 (25)	1 (2)	**<0.001**
obesity	0	0	5 (33)	0	3 (6)	**0.001**
dementia	0	0	3 (20)	2 (25)	0	**<0.001**
stroke	0	0	2 (13)	2 (25)	0	**0.001**
other cardiovascular disease	0	0	1 (7)	2 (25)	1 (13)	0.177
chronic kidney disease	0	0	1 (7)	0	0	0.284
cancer	0	0	1 (7)	0	0	0.284
HIV	0	0	0	0	3 (6)	0.551
Charlson Comorbidity Index *	0	0	2 (1–2)	4 (3–5)	0	**<0.001**

^a^ Data are expressed as median (interquartile range (IQR-25%-75%); ^b^ Acute symptoms reported by participants; ^c^ Symptoms on admission to hospital; NA: not applicable; (*) Bold values denote statistical significance (*p* < 0.05); For quantitative variables (age, days after symptom onset and Charlson comorbidity index) the non-parametric Kruskal–Wallis test was used. For categorical variables, the chi-square test or Fisher’s exact test was used, as appropriate.

**Table 2:** Area under the ROC curve of cytokines and chemokines to predict severity in SARS-CoV-2 infection.

Cytokine/Chemokine	ROC Area	95% CI
IFN-γ	0.164	0.041–0.287
TNF-α	0.200	0.071–0.329
IL-6	0.805	0.652–0.958
IL-10	0.678	0.526–0.831
CCL2/MCP-1	0.837	0.716–0.958
CXCL10/IP-10	0.752	0.574–0.876
